# Mapping the intracellular HMGB1 interactome and alterations induced by Toll-like receptor 4 activation

**DOI:** 10.1016/j.jbc.2025.110866

**Published:** 2025-10-27

**Authors:** Rebecka Heinbäck, Marit van der Zijde, Choi Har Tsang, Tilen Tršelič, Alexander Espinosa, Cecilia Aulin, Helena Erlandsson Harris

**Affiliations:** 1 Division of Rheumatology, Department of Medicine Solna, Karolinska Institutet, and Center for Molecular Medicine, Karolinska University Hospital, Stockholm, Sweden; 2 Broegelmann Research Laboratory, Department of Clinical Science, University of Bergen, Bergen, Norway

**Keywords:** HMGB1, BioID, interactome, TLR4, LPS, monocyte, proteomics, protein-protein interaction

## Abstract

The evolutionary conserved, mammalian protein HMGB1 is involved in chromatin-related mechanisms, autophagy, and sensing of immunogenic DNA. HMGB1 translocates from the nucleus to the cytosol in stressed myeloid cells, implicating functional changes. However, the role of intracellular HMGB1 in homeostatic conditions and alterations caused by stress remains largely unexplored. To better understand the intracellular roles of HMGB1, we defined the HMGB1 interactome in resting and LPS-stressed monocytic THP-1 cells using BioID-based proximity proteomics. We identified >100 proteins as parts of the HMGB1 interactome, where the majority were previously unknown interactors. Eight proteins significantly differed between resting and LPS-stressed cells: CACTIN, EIF4G3, GNA12, HSPB1, KOW domain-containing protein, MTHFD11, TADA2B and TPD52L2. Selected HMGB1 interactors were computationally docked to HMGB1, and interactions were confirmed *in vitro* by proximity ligation assays. Several proteins have implications in cell migration, which was verified experimentally in *HMGB1* knockout and shRNA-mediated knockdown cells. HMGB1 deficiency led to an increase in migration compared to wild-type cells or scrambled shRNA control. Furthermore, larger cell size and dysregulated actin polymerization were evident in these cells. In conclusion, we have for the first time identified the intracellular HMGB1 interactome and could identify several HMGB1-protein interactions. Our results reveal previously undescribed homeostatic intracellular roles of HMGB1 in addition to changes caused by cell stress. Our study forms a gateway for future research on HMGB1 functions, both as a pivotal protein for mammalian cell homeostasis and in cellular stress responses.

The evolutionary conserved protein High mobility group box 1 (HMGB1) is a multifunctional protein with distinct roles in different cellular compartments. Mice deficient in HMGB1 are born malformed and die within 24 h after birth, and therefore HMGB1 is essential for life (1). In healthy cells, HMGB1 is predominantly present in the nucleus where it binds to chromatin to promote genome stability, regulate DNA transcription, and control recombination and DNA repair (2, 3, 4). In addition to its nuclear role, HMGB1 can be secreted into the extracellular space, where it acts as an alarmin (5, 6). This makes HMGB1 a potential therapeutic target in several diseases, including rheumatoid arthritis, cancer and sepsis (7, 8). In response to cellular stress, increased levels of HMGB1 are found in the cytosol (9, 10). Cytosolic HMGB1 has been implicated in autophagy and Toll-like receptor 9 (TLR9)-sensing of immunogenic DNA (11, 12, 13). Following cytosolic translocation, HMGB1 interacts with Beclin-1, leading to dissociation between Beclin-1 and the anti-apoptotic protein BCL2, thereby promoting autophagy and cell survival (14). Cytosolic HMGB1 has also been implicated in lipopolysaccharide (LPS)-induced inflammatory stress, where it was demonstrated to interact with NOD2 and ATG16L1 to mediate the formation of autophagosomes in microglia (15). However, compared to nuclear and extracellular functions of HMGB1, its cytosolic features are understudied. To delineate the intracellular functions of this highly conserved and essential protein and to aid the development of future HMGB1-targeting therapeutics, we set out to reveal the interactors of HMGB1 during homeostasis and following TLR4-induced cell stress by LPS. To this end, we compared the HMGB1 interactomes in resting and LPS-stressed monocytic THP-1 cells using BioID-based proximity proteomics (16, 17), followed by validation of individual hits using computational protein-protein docking and proximity ligation assay. Finally, we performed functional assays showing the importance of HMGB1 in cellular migration. We identified distinct changes in the HMGB1 interactome after LPS stress, thus providing new insights into the molecular mechanisms of HMGB1 in both resting and stressed cells.

## Results

### The HMGB1 interactome changes following TLR4 activation and is enriched for proteins involved in DNA transcription, signal transduction, and mitochondrial function

**Figure 1:**
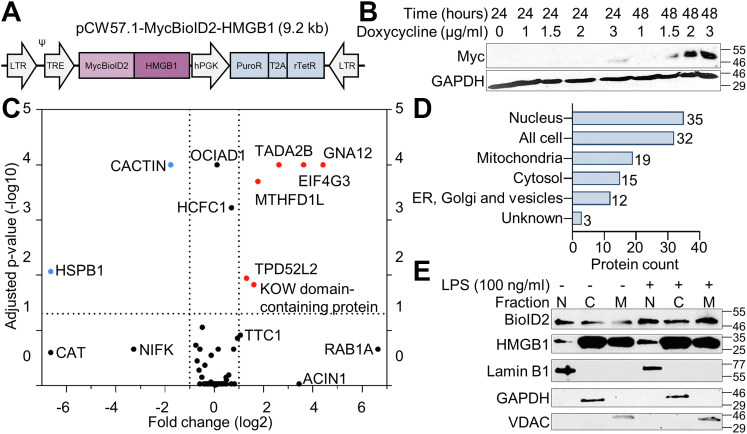
**The HMGB1 interactome in THP-1 cells changes after TLR4 activation.***A*, illustration of construct used to insert MycBioID2-HMGB1 into THP-1 cells. *B*, titration of doxycycline concentration to induce MycBioID2-HMGB1 expression. Lysates were analyzed by Western blotting using anti-Myc-HRP. GAPDH was used as loading control. *C*, Volcano plot of the HMGB1 interactome in resting (n = 3) and LPS-stimulated (n = 3) THP-1 cells. *p*-values were calculated by multiple paired two-way ANOVA corrected for multiple testing by FDR. *D*, the subcellular localization of proteins in the HMGB1 interactome. *E*, lysates from resting and LPS stressed THP-1 cells were fractioned into nuclear (N), cytosolic (C) and mitochondrial (M) fractions and analyzed by Western blotting using anti-HMGB1 and anti-BioID2. LaminB1, GAPDH and VDAC were used as loading controls (n = 3). All Western blots have been marked with molecular weights (kDa) on the right side.

To identify the HMGB1 interactome in resting and LPS-stressed cells, we performed BioID-based proximity proteomics in monocytic THP-1 cells. Briefly, we induced expression of MycBioID2-HMGB1 (MW = 55 kDa) using doxycycline (Fig. 1, *A* and *B*) followed by addition of biotin. To enable relative quantification of the interactome with and without LPS stimulation, we used stable-isotope labeled amino acids. We first verified that cellular biotinylation was increased after doxycycline-induced MycBioID2-HMGB1 expression and addition of biotin (Fig. S1, *A* and *B*), and then confirmed successful LPS activation by measuring secretion of IL-8 in the supernatants (Fig. S1*C*). To rule out potential interference caused by overexpression or by the fusion to BioID2, we performed.

Western blotting (Fig. S1*E*), immunocytochemistry (Fig. S1*F*) and lysate fractioning (Fig. 1*E*). The MycBioID2-HMGB1 expression was low compared to endogenous HMGB1 and localized in the same subcellular compartments in both resting and LPS-stressed cells. However, lysate fractioning (Fig. 1*E*) shows different distributions of endogenous HMGB1 and MycBioID2-HMGB1, whereas immunocytochemistry (Fig. S1*E*) displays a high degree of co-localization (r_PBS_ = 0.83, r_LPS_ = 0.75, Fig. S1*G*) corresponding to 75% and 83% of MycBioID2-HMGB1 co-localizing to endogenous HMGB1 in resting and LPS-stressed cells, respectively (Fig. S1*H*). Also, immunocytochemistry shows a strong nuclear signal and LPS-induced cytosolic translocation of both endogenous HMGB1 and MycBioID2-HMGB1. A potential explanation for the deviating result after lysate fractioning is likely due to leakage of small nuclear proteins (MW_HMGB1_ = 29 kDa) into the cytosolic fraction during the fractionation procedure. Following 24 h of BioID2-mediated biotinylation of the interactome, biotinylated proteins were extracted and identified by LC-MS/MS, which resulted in 116 proteins being defined as part of the HMGB1 interactome (data Table S1-S2). We identified eight proteins with significant abundance differences between resting and LPS stressed cells (Fig. 1*C*). During LPS stress, there was an increased abundance of EIF4G3, GNA12, KOW domain-containing protein, MTHFD1L, TADA2B and TPD52L2, and a decreased abundance of CACTIN and HSPB1. Classification of the proteins based on their reported subcellular localization revealed that HMGB1 interacts with proteins in multiple subcellular localizations, including the nucleus, cytosol, mitochondria, ER-Golgi and vesicles (Fig. 1*D*). The HMGB1 interactome included 35 proteins solely located in the nucleus and 32 proteins located both in the cytosol and in the nucleus. The presence of HMGB1 in the nuclear, cytosolic and mitochondrial compartments was confirmed in both resting and LPS-stressed THP-1 cells by Western blots of lysate fractions (Fig. 1*E*).

**Figure 2:**
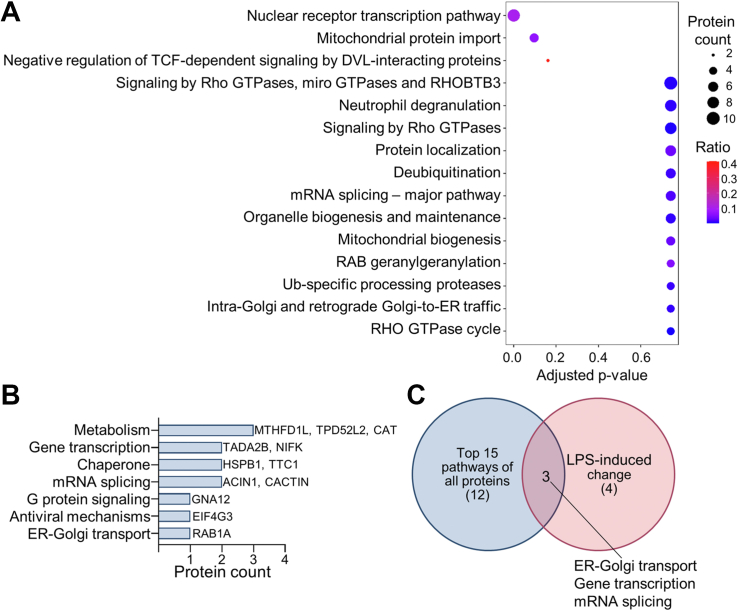
**HMGB1 interacts with proteins involved in nuclear receptor transcription, mitochondrial protein import and DVL-mediated signaling.***A*, top 15 pathways of reactome enrichment analysis where adjusted *p*-value is displayed on the x-axis. Dot size relates to the number of proteins identified in the pathway and color represents the ratio of proteins in the interactome and the total number of proteins in the pathway. *B*, pathways of differentially abundant proteins following LPS stress. *C*, Venn diagram showing the overlap between the top 15 pathways of all proteins and the pathways of the LPS-induced changes in the HMGB1 interactome.

To determine the intracellular processes where HMGB1 is involved, proteins in the HMGB1 interactome were analyzed by Reactome enrichment analysis (Fig. 2*A*, data Table S2). The top three pathways were “Nuclear receptor transcription pathway”, “Mitochondrial protein import” and “Negative regulation of TCF-dependent signaling by DVL-interacting proteins”. Involvement of HMGB1 in the nuclear receptor transcription pathway has been well-characterized (4). The interactome analysis also revealed two cytosolic signaling transduction pathways, including Wnt signaling (“Negative regulation of TCF-dependent signaling by DVL-interacting proteins”) and Rho GTPase signaling (“Signaling by Rho GTPases, miro GTPases and RHOBTB3” and “Signaling by Rho GTPases”). Both Wnt and Rho GTPase signaling are novel to the intracellular functions of HMGB1. Reactome enrichment analysis was further used to identify the roles of the proteins having different abundance in resting and LPS-stressed cells (Fig. 2*B*, data Table S2). Four of the seven identified pathways were unique for LPS-stressed cells, namely metabolism, chaperone-related activity, G-protein-mediated signaling and antiviral mechanisms (Fig. 2, *B* and *C*).

### Computational docking reveals direct interaction between HMGB1 and proteins in the interactome

**Figure 3:**
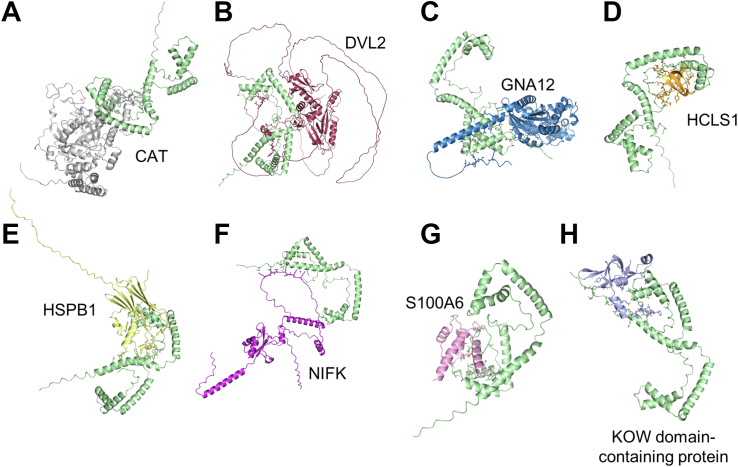
**Eight HMGB1-protein models showed acceptable docking.** Images of HMGB1-protein complexes following computational docking. HMGB1 is shown in green docked to (*A*) CAT, (*B*) DVL2, (*C*) GNA12, (*D*) HCLS1, (*E*) HSPB1, (*F*) NIFK, (*G*) S100A6, and (*H*) KOW domain-containing protein.

To assess direct interactions between HMGB1 and proteins in the interactome, computational docking was performed using the high-throughput software InterEvDock3. Proteins with abundances altered by LPS stress (−1.0 ≤ log_2_ fold change (LPS/PBS) ≥ 1.0) were docked to HMGB1. EIF4G3 was excluded as it is part of the protein translation machinery. HCLS1, S100A6, DVL1, and DVL2 did not display altered abundance between resting and stressed cells but were docked to HMGB1 based on their reported intracellular roles in cellular stress and immunity (Table S2). Eight out of sixteen proteins showed comparable docking properties to the known interactors NR3C1 and MD2 (Table 1, Table S3). Images of the acceptable models are presented in Figure 3 (incl. CAT, DVL2, GNA12, HCLS1, HSPB1, KOW domain-containing protein, NIFK and S100A6). Based on the top five interacting amino acids in the complexes, protein interactions were identified in all HMGB1 domains (Table S4, Fig. S2). The known interaction partners NR3C1 and MD2 were defined to bind in the A-box region of HMGB1. MD2 has previously been reported to have a functionally important interactor site in the B-box region and to bind in the A-box region (18, 19). The exact site of interaction between HMGB1 and NR3C1 has to our knowledge never been described. In addition, DVL2 and GNA12 were predicted to bind within the A-box region. In total, four proteins have their predicted interaction sites within the B-box region proximal to the redox sensitive Cys106 (incl. HCLS1, S100A6, HSPB1, and CAT). Finally, NIFK and KOW domain-containing protein were predicted to bind the acidic c-tail region.

**Table 1:** RMSD scores of successfully docked HMGB1-protein interactions

	Proteins	*i*_rms_ (Å)	*L*_rms_ (Å)	*f*(nat)	CAPRI Ranking
Increased by LPS	KOW domain-containing protein	0.09	1.25	0.58	High
	GNA12	2.43	8.30	0.35	Acceptable-Medium
No abundance change	HCLS1	0.25	1.81	0.39	Medium-High
	S100A6	0.10	5.29	0.72	Medium
	DVL2	9.59	2.90	0.57	Acceptable
Decreased by LPS	HSPB1	2.01	2.02	0.9	Medium
	NIFK	0.56	3.40	0.76	Medium-High
	CAT	2.57	7.67	0.29	Acceptable
Comparison to known HMGB1 protein interactions	NR3C1	1.98	5.32	0.79	Medium
	MD2	1.24	3.197	0.81	Medium-High

I_RMS_ - The root mean square deviation (RMSD) of amino acids at the interface. L_RMS_ – The overall RMSD of entire complex. F(nat) – the fraction of correct contacts at the interface (within 5 Å). CAPRI ranking is the official ranking system summarizing all three values ranging from acceptable – medium – high.

### HMGB1 interacts with all successfully docked proteins in both resting and LPS-stressed THP-1 cells

**Figure 4:**
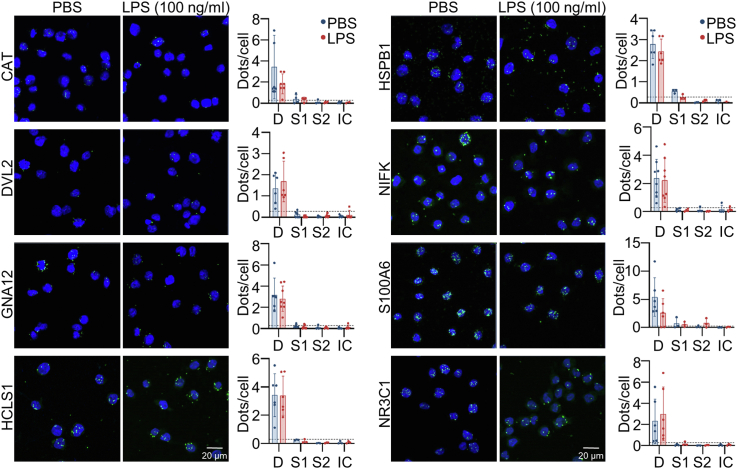
**HMGB1 interacts with all successfully docked proteins.** PLA was performed on resting (n = 6–8) and LPS-stressed (n = 6–8) THP-1 cells to validate the interaction of endogenous HMGB1 with proteins previously indicated to interact with HMGB1 *in silico*. Representative images of PLA between HMGB1 and CAT, DVL2, GNA12, HCLS1, HSPB1, NIFK, and S100A6. NR3C1 was used as a positive control. *Green* fluorescent spots indicate interactions. Nuclei are shown in *blue* (DAPI). Scale bars represent a distance of 20 μm. Interaction was quantified as dots/cells in double stained (D), anti-target-stained (S1), anti-HMGB1-stained (S2), and irrelevant-stained (IC, normal rabbit IgG and normal mouse IgG2b) cells. The lowest limit of detection was determined for each target as the average + 2 × standard deviation of all controls (y-axis line). Data is shown as individual data points and average ± standard deviation. Data were analyzed by comparing the double-stained resting and LPS-stressed cells using Mann–Whitney *U* tests. All *p*-values >0.05 are considered as non-significant.

To validate interaction between HMGB1 and selected proteins within its interactome, proximity ligation assay (PLA) was performed on endogenous proteins in resting and LPS-stressed THP-1 cells. PLA immunostainings were performed for HMGB1 and proteins showing acceptable docking results. The KOW domain-containing protein was excluded due to no commercially available antibodies. The PLA data demonstrated interaction between HMGB1 and all investigated proteins (incl. CAT, DVL2, GNA12, HCLS1, HSPB1, NIFK, S100A6, and NR3C1) indicated by an increase in green fluorescent spots compared to the negative controls (Figs. 4 and S3*B*). All proteins except DVL2 displayed equal signals compared to the positive control NR3C1 (glucocorticoid receptor). Even though the LC-MS/MS data showed differences in HMGB1-protein interaction following LPS stress (CAT, GNA12, HSPB1, and NIFK, Figs. S3*A* and 1*C*), there were no significant differences in PLA spots/cell between resting and LPS-stressed cells at the investigated time point (Fig. 4).

### HMGB1 regulates cell migration and actin polymerization

**Figure 5:**
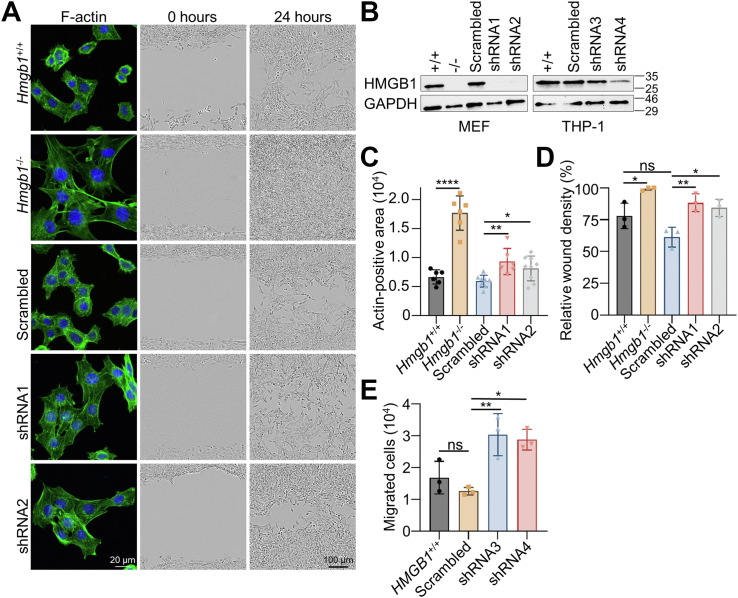
**HMGB1 regulates actin polymerization and cell migration.***A*, *Hmgb1* knockout and shRNA-induced knockdown MEFs were stained for actin (*green*) and DAPI (*blue*). Scale bar represent a distance of 20 μm. Cells were used in wound healing assay and images were taken at 0 and 24 h (scale bar represent a distance of 100 μm). *B*, *Hmgb1* knockout and shRNA-induced knockdown in MEFs and THP-1 cells were confirmed by Western blot against HMGB1 where GAPDH was used as a loading control. *C*, actin-positive area was quantified (pixels) and normalized to the cell number (n = 6–8). *D*, wound-healing assays were analyzed by quantifying the relative wound density (%) after 24 h (n = 3). *E*, wild-type and shRNA-transduced THP-1 cells (n = 3) were cultured in trans-well chambers for 5 hours before quantification of cells in the lower chamber. All data are shown as individual data points and average ± standard deviation. Data were analyzed by ordinary one-way ANOVA, and *p*-values were corrected for multiple testing using Two-stage step-up method of Benjamini, Krieger and Yekutieli (*C*) or Tukey (*D*–*E*). *p*-values are represented as ∗ where ∗ < 0.05, ∗∗ < 0.01, ∗∗∗ < 0.001, and ∗∗∗∗ < 0.0001.

As several HMGB1-interacting proteins are involved in actin polymerization (incl. GNA12, HCLS1, HSPB1 and S100A6. See Table S2), we performed actin stainings in *Hmgb1* wildtype and knockout mouse embryonic fibroblasts (MEFs) (Figs. 5, *A* and *B* and S4*A*). HMGB1 deficient cells were significantly larger than wildtype cells determined by actin-positive area per cell (Fig. 5*C*). A similar phenotype was also detected following induced *Hmgb1* knockdown by shRNA in wild-type cells (Figs. 5, *A*–*C* and S4*A*). Since actin is important for migration, wound healing assays were performed where *Hmgb1-deficient* cells migrated at a significantly faster rate compared to wild-type cells and/or scrambled shRNA-control (Figs. 5, *A*, *D* and S4*B*). To validate these results in THP-1 cells, cells were transduced with *HMGB1*-targeting shRNA (Fig. 5*B* and S4*A*) and used in trans-well assays. *HMGB1-*deficient cells displayed an increased migration through the membrane to the bottom chamber compared to the scrambled shRNA control (Fig. 5*E*). Taken together, loss of HMGB1 in both MEFs and THP-1 cells leads to increased migration and dysregulation of actin polymerization.

## Discussion

HMGB1 is a multifunctional protein involved in homeostasis, immune defense as well as disease pathogenesis. It is therefore considered a promising target for future therapeutics. Here, we have identified the intracellular interactome of HMGB1 in human monocytic THP-1 cells and its alterations during resting and LPS-TLR4-induced stress conditions. The DNA-binding properties of HMGB1 are well-characterized (6, 20, 21). In line with prior literature, our data suggest nuclear receptor transcription to be the most prominent pathway of the HMGB1 interactome. Our data further revealed that HMGB1 interacts with proteins in all subcellular compartments and eight proteins significantly differ in their interactions with HMGB1 following LPS-induced stress. We could validate seven of these novel endogenous HMGB1-protein interactions in cells using proximity ligation assay and predicted their binding to different HMGB1 domains *in silico*. However, we did not detect quantitative differences between resting and LPS-stressed cells by proximity ligation assay at the selected time point. Nevertheless, it is difficult to compare BioID to the proximity ligation assay since BioID captures 24 h of proximity labeling in live cells, whereas the proximity ligation assay is a snapshot of one selected time point. Furthermore, several of the identified proteins affect actin polymerization, and we demonstrated that loss of HMGB1 in THP-1 cells and in MEFs causes alterations in actin polymerization and increased cell migration.

Enrichment analysis of the entire HMGB1 interactome suggests a role of HMGB1 in Wnt signaling (“Negative regulation of TCF-dependent signaling by DVL-interacting proteins”) and Rho GTPase signaling (“Signaling by Rho GTPases, miro GTPases and RHOBTB3” and “Signaling by Rho GTPases”). Following LPS stress, increased interaction between HMGB1 and GNA12 was detected suggesting a direct involvement of HMGB1 in Gα_12/13_ signaling after TLR4 activation. So far, there is no evidence supporting the involvement of HMGB1 in intracellular G-protein coupled receptor signaling. Gα_12/13_ proteins are known to be coupled to over 30 G-protein coupled receptors and have diverse downstream effects, including cell migration (22). In addition, our data demonstrated that HMGB1 interacts with HCLS1 and ARPC3, two proteins known to be essential for actin polymerization (23, 24). HMGB1 has furthermore been reported to be involved in migration and invasion of gastric cancer cells (25). Taken together, the interactome analysis implicated a regulatory role for HMGB1 in cell motility and migration. This was proven experimentally as loss of HMGB1 in MEFs caused dysregulation of actin polymerization and increase in cell migration, and loss of HMGB1 in THP-1 cells led to increased cell migration in trans-well assays. HMGB1 has been associated to filopodia formation in neuronal cells, further strengthening our hypothesis of a direct involvement in cytosolic actin polymerization (26).

Interestingly, Gα_12/13_ have been suggested to act downstream of Frizzled 4, leading to cytoskeletal rearrangement and Rho signaling (27). Based on the LC-MS/MS data, the interaction between Gα_12/13_ (GNA12) and HMGB1 increased following LPS stress, implying that the role of HMGB1 in actin rearrangement is affected by cell stress. Nevertheless, future studies are warranted to address the molecular mechanisms of HMGB1-mediated actin polymerization, the impact of GPCR signaling and their changes during cell stress.

There are certain limitations to our study. All experiments were performed using cell lines and have not been confirmed in primary cells. As the fusion of BioID2 and HMGB1 is double the size of endogenous HMGB1, the biological functions of HMGB1 could be affected. We could detect small differences in subcellular distribution by lysate fractioning and immunocytochemistry. Also, co-immunoprecipitation of endogenous proteins would strengthen the study by proving a direct protein interaction. Nevertheless, we confirmed the interaction with the glucocorticoid receptor, a well-known HMGB1-protein interaction, identified several proteins involved in the nuclear receptor transcription pathway and showed a functional role of HMGB1 in actin polymerization and cell migration.

Taken together, this study presents the first defined HMGB1 interactome and reveals changes in response to LPS-TLR4-induced stress. Based on the identified proteins, we could map HMGB1 to all subcellular compartments and associate it with multiple intracellular mechanisms, including both known and previously unrecognized functions. Together with docking models and proximity ligation assays, we have demonstrated seven novel HMGB1-protein interactions involved in both nuclear and cytosolic processes. In line with previous studies, we showed that HMGB1 is involved in actin polymerization and cell migration and added molecular details of its involvement. Finally, our study directs future molecular HMGB1 research areas to clarify the vital, homeostatic functions of intracellular HMGB1 and its involvement in cellular stress responses. It also suggests intracellular drug targets for future HMGB1-therapeutics.

## Experimental procedures

### Cloning of pCW57.1-MycBioID2-HMGB1

The coding sequence of HMGB1 was amplified using pETM-11-HMGB1 as a template and the following primers with XhoI and SalI restriction sites added at the 5′ and 3′ end, respectively: CAACAACTCGAGGGTGGAGGCGGGTCTATGGGCAAAGGAGATCCTAAG and CAACAAGTCGACTTATTCATCATCATCATCTTCTTCTTCATCTTC. Additionally, a linker (15 bp) was added between the cleaving site of XhoI and the HMGB1 sequence (see supporting information). PCR reaction was initiated with denaturation (95 °C, 5 min), followed by 30 cycles of denaturation (95 °C, 15 s), annealing (60 °C, 15 s) and elongation (72 °C, 1 min). The PCR product was thereafter inserted into pCR-Blunt II-TOPO (Invitrogen) according to the manufacturer’s protocol. The sequence of HMGB1 was extracted and inserted at the restriction enzyme sites of XhoI and SalI in pBABE-puro-MycBioID2 (Addgene, #80900). Finally, the sequence of MycBioID2-HMGB1 was extracted at the sites of EcoRI and SalI and inserted at the sites of NheI and SalI in pCW57.1 (Addgene, #41393).

### Cell cultures

*Hmgb1* wildtype and knockout mouse embryonic fibroblasts (MEFs) were kindly provided by M.E. Bianchi(1). MEFs and human embryonic kidney 293 (HEK293) cells were cultured in DMEM (Sigma Aldrich). THP-1 (human acute leukemia-derived monocyte) cells were cultured in RPMI-1640 (Sigma Aldrich). All cell cultures were supplemented with fetal calf serum (10%), penicillin (100 U/ml) and streptomycin (100 μg/ml). Cells were cultured at 37 °C in a humidified atmosphere containing 5% CO_2_.

### Lentiviral packaging and viral transduction

3 × 10^5^ HEK293T were seeded per well in a 6-well plate. Mixtures of 75 μl Opti-MEM I Reduced serum medium (Thermofisher Scientific), 3 μl X-tremeGENE 9 DNA Transfection reagent (Sigma Aldrich), 500 ng transgene-coding plasmid, 250 ng pMD2.G (Addgene, #12259) and 250 ng psPAX2 (Addgene, #12260) were incubated (15 min, RT) and added dropwise to cells. The following plasmids were used: pCW57.1-MycBioID2-HMGB1, mouse *Hmgb1*-targeting shRNA plasmids (Sigma Aldrich, TRCN0000365912 (no.1) and TRCN0000374092 (no.2)), human *HMGB1*-targeting shRNA plasmids (Sigma Aldrich, TRCN0000018932 (no.3) and TRCN0000018934 (no.4)) and TRC2-pLKO-puro non-target shRNA (Sigma Aldrich, SHC202). Viral packaging was conducted for 4 days before harvesting (500g, 5 min). 7.5 × 10^5^ THP-1 cells or 3 × 5 × 10^5^ MEF^Hmgb1+/+^ were seeded per well in a 6-well plate and infected with harvested virus. Polybrene (2.5 μg/ml, Sigma Aldrich) was used to enhance transduction efficiency. Centrifugal inoculation (1400g, 1 h) was applied to THP-1 cells. Transduced cells were selected by the addition of puromycin (2.5 μg/ml, Thermofisher Scientific) to the cell media.

### Validation of HMGB1 knockdown

Knockdown efficiency was estimated using qPCR and confirmed by Western blot. RNA was extracted using the RNeasy Plus Micro Kit (Qiagen) according to the manufacturer’s instructions. Reverse transcription to cDNA was done using the iScript cDNA synthesis kit (Bio-Rad) according to the manufacturer’s instructions. qPCR was performed using the KiCqStart SYBR Green qPCR ReadyMix (Sigma Aldrich) and run on a CFX384 Thermal Cycler according to the manufacturer’s instructions. The primer pairs are specified in Table S1. To calculate the ddCt values, Ct data were normalized against human *GAPDH* or mouse *Hprt*.

### SDS-PAGE and western blot

4 to 20% gradient Tris-Glycine gels (Bio-Rad) were loaded with equal amounts of protein (10–25 μg) and run at 110V for 1 h. Proteins were transferred to nitrocellulose blotting membranes (0.45 μm, GE Healthcare) at 100 V for 1 h. Membranes were blocked in 5% dry milk-TBST at RT for 1 h followed by incubation with HRP-conjugated primary antibodies or streptavidin-HRP (GE Healthcare, 1:10,000) at RT for 1 h. Incubation with non-conjugated primary antibodies was conducted at 4 °C o/n, followed by incubation with HRP-conjugated secondary antibodies (RT, 1 h). ECL-chemiluminescence was detected by ChemiDoc MP imaging system (Bio-Rad) or by exposing the membranes to X-Ray films (Amersham, GE Healthcare). The following antibodies were used: 2040 (Mouse monoclonal-HRP against Myc-tag, Cell signaling technology), 3683 (Rabbit monoclonal-HRP against GAPDH, Cell signaling technology), 2G7 (Mouse monoclonal IgG2b against HMGB1 (28)), ab232733 (Mouse monoclonal IgG1 against BioID2, Abcam), 15068 (Rabbit monoclonal-HRP against LaminB1, Cell signaling technology), 4866 (Rabbit polyclonal against VDAC, Cell signaling technology), 7076 (affinity-purified horse anti-mouse IgG-HRP conjugate, Cell signaling technology) and 170-6515 (Goat anti-Rabbit IgG (H + L)-HRP-conjugate, Biorad). Antibody specificity: 2G7 has been knockout-validated by us, and ab232733 was validated by induced expression of BioID2. Antibodies used for loading control were not tested.

### Immunocytochemistry

5 × 10^5^ THP-1 or THP-1^MycBioID2-HMGB1^ cells were seeded into each well in a 6-well plate (n = 3). THP-1^MycBioID2-HMGB1^ cells were treated with doxycycline (2 ug/ml) for 1.5 days. Thereafter, cells were stimulated overnight with LPS (100 ng/ml, 127K Sigma Aldrich) or PBS as a control. Cells were fixed in 4% paraformaldehyde (RT, 15 min) and dried onto gelatin-coated microscope slides. Cells were permeabilized by 0.2% Triton-X100 (RT, 15 min) and blocked using 10% normal goat serum (DAKO, RT, 1 h). HMGB1 and BioID2 were stained using rabbit monoclonal IgG against HMGB1 (ab79823, Abcam) and mouse monoclonal IgG1 against BioID2 (ab232733, Abcam). Primary antibodies were detected by Goat anti-Mouse IgG, IgM (H + L) Secondary Antibody, Alexa Fluor 488 (Thermofisher Scientific, A-10680) and Goat anti-Rabbit IgG (H + L) Secondary Antibody, Alexa Fluor 594 (Thermofisher Scientific, A-11012). Nuclei were stained by DAPI (RT, 5 min) before slides were mounted using ProLong gold antifade mountant (Thermofisher Scientific). Slides were imaged in an LSM 880 confocal microscope (Carl Zeiss, Jena, Germany) at 63× magnification. ZEN 2.3 SP1 FP3 (black) was used to obtain images. All conditions were run in three separate wells to obtain n = 3. Antibody specificity: ab79823 has been knockout-validated by Abcam, and ab232733 was validated by induced expression of BioID2. Pixel-by-pixel correlation was performed in CellProfiler (4.1.3) to obtain Pearson’s correlation coefficients and Mander’s overlap coefficients for each cell. In total, 71 resting and 55 LPS-stressed THP-1s were analyzed (n = 3).

### Lysate fractionation of THP-1 cells

2.25 × 10^6^ THP-1^MycBioID2-HMGB1^ cells were seeded into T-25 flasks (n = 3). Cells were treated with doxycycline (2 ug/ml) for 1.5 days. Thereafter, cells were stimulated overnight with LPS (100 ng/ml, 127K Sigma Aldrich) or PBS as a control. Fractionation of nuclear, cytosolic and mitochondrial fractions were done according to the manufacturer’s protocol (Abcam, ab109719). Purity of fractions was assessed by Western blot against LaminB1 (nuclear), GAPDH (cytosol), and VDAC (mitochondria). All conditions were run in three separate flasks to obtain n = 3.

### ELISA

Secreted IL8 was measured using Human IL-8/CXCL8 DuoSet ELISA (R&D Systems) according to kit instructions.

### SILAC labeling of THP-1 cells

Stable isotype labeling by amino acids in cell culture (SILAC) was done to transduced THP-1 cells before BioID. THP-1 cells were incubated in light or heavy media for 1 week, whereof light media contained L-Arginine and L-Lysine and heavy media contained ^13^C_6_^15^N_2_ L-Lysine and ^13^C_6_^15^N_2_ L-Arginine. SILAC-labeled cells were cultured with the supplementation of dialyzed fetal calf serum (10%), penicillin (100 U/ml) and streptomycin (100 μg/ml). Based on the heavy:light protein distribution, the degree of heavy amino acid incorporation was estimated to be >88% (Fig. S1*D*). Thereafter, 6 × 10^5^ cells/ml were seeded and MycBioID2-HMGB1 expression was induced with 2.0 μg/ml doxycycline (Sigma Aldrich) for 48 h. Thereafter, doxycycline was removed, and heavy-labeled cells were stimulated with LPS (100 ng/ml, 127K, Sigma Aldrich) for 1 h before biotin was added (50 mM, Sigma Aldrich, 24 h). Cells cultured with light amino acids were used as a control and only treated with biotin. The conditions were set up in three separate T-175 flasks (n = 3). The experiments were performed in parallel to avoid batch differences caused by different LC-MS/MS runs and/or different freezer storage durations.

### BioID-based proximity labeling

After stimulation, cells were lysed and the biotinylated proteins were extracted as reported previously (16). Briefly, cells were treated with lysis buffer containing 50 mM Tris-Cl (pH 7.4), 500 mM NaCl, 0.2% SDS, with the addition of Halt Protease and Phosphatase Inhibitor Cocktail (Thermo Scientific) and 1 mM DTT. After completion of lysis, protein concentrations were determined by Bradford assay and the lysates containing heavy and light proteins were pooled (1:1). The pooled lysate was incubated with Dynabeads MyOne Streptavidin C1 (Invitrogen) (o/n, 4 °C) before pull-down. After washing, the beads were collected by centrifugation (2000*g*, 5 min, RT) and suspended in ammonium bicarbonate (50 mM). An aliquot was saved for Western blot analysis against biotin to confirm protein biotinylation. The remaining samples were sent for LC-MS/MS analysis.

### On-beads digestion

Following magnetization of 50 μl streptavidin-activated beads, the beads were washed twice with ammonium bicarbonate (50 mM, pH 8). Samples were incubated in a shaker (300 rpm, 30 min, RT) followed by centrifugation and magnetization. Before proteolytic digestion, beads were resuspended in 70 μl ammonium bicarbonate (50 mM, pH 8) with the addition of DTT (20 mM, Sigma Aldrich) and proteins reduced at 37 °C for 45 min. Thereafter, cysteines were reduced by iodoacetamide (IAA, 20 mM, Sigma Aldrich) at room temperature for 30 min. Digestion was performed by the addition of 4 μg sequencing grade modified trypsin (Promega, 16 h, 37 °C, 300 rpm). Samples were magnetized for 30 s and supernatants transferred to new tubes, followed by further digestion using 1 μg of trypsin (3 h, 37 °C) and magnetized again (30 s). Supernatants were saved, and digestion stopped by the addition of formic acid at a final concentration of 5%. Samples were cleaned using a C-18 HyperSep plate (Thermo Scientific) and dried in a SpeedVac.

### LC-MS/MS analysis

Chromatographic separations of peptides were performed on a 50 cm EASY-spray column over a linear 90 min gradient at 300 nl/min flow rate using a nanoUltimate 3000 system (Thermo Fisher Scientific) coupled to an Orbitrap Fusion mass spectrometer (Thermo Scientific). The gradient went from 2 to 26% of buffer B (2% acetonitrile, 0.1% formic acid) in 90 min and up to 95% of buffer B in 2 min. Eluted peptides were ionized by electrospray ionization and the survey MS spectra were acquired at the resolution of 120,000 in the range of m/z 350 to 1700. MS/MS data were obtained with 30% normalized collision energy using higher-energy collisional dissociation (HCD) for ions with charge z > 1 at a resolution of 30,000 and first mass at 100.

### Processing of LC-MS/MS raw data

The raw data files were directly loaded in Proteome Discoverer v2.3 and searched against the human SwissProt protein database (42,252 entries) and the most common contaminants (244 entries) using the Mascot 2.5.1 search engine (Matrix Science Ltd), targeting 1% false discovery rate (FDR). Parameters were chosen as follows: up to two missed cleavage sites for trypsin, precursor mass tolerance 10 ppm, and 0.05 Da for the HCD fragment ions. Dynamic modifications of oxidation on methionine, deamidation of asparagine and glutamine and SILAC Arg10/Lys8 were set. For quantification, both unique and razor peptides were requested. To compare protein expression fold changes pairwise protein ratio was calculated.

### Data processing and statistical analysis

To filter the data, all ribosomal proteins were excluded, as well as proteins scoring >100/400 according to CRAPome V1.0 (29). Also, hits obtained in only one of the three data sets were excluded. Following filtering, 116/536 (22,05%) proteins remained. Missing data was imputed as half of the minimum in the respective group. Data was considered normally distributed since it passed the D'Agostino & Pearson test and therefore analyzed using parametric tests. *p*-values were calculated by 2-way ANOVA, where values were matched between the non- and LPS-stimulated, pooled samples. Correction for multiple testing was done according to the false discovery rate (FDR). *p*-values < 0.05 were considered as significant (Graphpad Prism 8.4.3). HMGB1-BioID hits were further sorted based on their subcellular localization. If possible, subcellular localization was based on what is published in the Human Protein Atlas project (12). If not possible, Uniprot was used (13).

### Reactome analysis

The proteins of the interactome were put into a pathway analysis in Reactome (30), including IntAct data (31). The top significant pathways of all proteins were identified. Among the protein interactions changed by LPS, Reactome was used to identify their involvement in cellular processes. Dot plots were done in RStudio© (2021.09.2). *p*-values in the enrichment analysis were considered to be of low importance since individual proteins are normally not found in several steps of the same pathway, leading to a low number of identified proteins in one pathway.

### Computational docking of HMGB1-protein interactions

All BioID hits with a higher log_2_ fold change than 1.0 and lower than −1.0 were computationally docked to HMGB1. In addition, HCLS1, S100A6, DVL1 and DVL2 were docked to HMGB1. All proteins were docked using the high-throughput software InterEvDock3 (32). Structural protein data of the hits were obtained from AlphaFold (33, 34) in PDB format. Thereafter, the top Frodock model of HMGB1-protein complexes were scored based on root mean square deviation (RMSD) of the interface (I_rms_) and ligand (L_rms_), as well as the f(nat) in PyMOL (V1.7.4). The success of docking was ranked according to CAPRI criteria of protein-protein modeling (35).

### Proximity ligation assay

1 × 10^6^ THP-1 cells were seeded in a 6-well plate and rested for 4 to 5 h. Thereafter, half of the wells were stimulated with LPS (100 ng/ml, 127K, Sigma Aldrich) o/n before fixing in paraformaldehyde (4%, RT, 15 min). Cells were dried onto poly-L-Lysine-coated microscope slides and thereafter permeabilized with 0.2% Triton-X100 (RT, 15 min). Proximity ligation assay was performed using the Naveniflex MR kit (Navinci) according to the manufacturer’s instructions. Briefly, cells were blocked by the addition of 5% normal goat serum (1 h, 37 °C). Binding of primary antibodies was performed at 4 °C o/n. Finally, secondary antibodies were bound followed by PCR reaction (37 °C). All washes were done in 37 °C. Nuclei were stained with DAPI (Invitrogen, 10 min, RT) before slides were mounted using ProLong gold antifade mountant (Thermofisher Scientific). Slides were imaged in an LSM 880 confocal microscope (Carl Zeiss, Jena, Germany) at 63× magnification. ZEN 2.3 SP1 FP3 (black) was used to obtain images. For all experiments, staining with single antibodies as well as irrelevant rabbit IgG and irrelevant mouse IgG2b were done as negative controls. The following primary antibodies were used: 2G7 (monoclonal mouse IgG2b against HMGB1, in-house produced), 12980 (monoclonal rabbit IgG against CAT, Cell signaling technology), 95357 (monoclonal rabbit IgG against HSP27 (HSPB1), Cell signaling technology), ab181975 (recombinant rabbit IgG against S100A6, Abcam), 4503 (polyclonal rabbit IgG against HCLS1, Cell signaling technology), PA5-83283 (polyclonal rabbit IgG against NIFK, Thermofisher scientific), ab236617 (polyclonal rabbit IgG against GNA12, Abcam), 3216 (polyclonal rabbit IgG against DVL2, Cell signaling technology). Normal rabbit IgG (Dako) and normal mouse IgG2b (Dako) were used as controls. All conditions were run in two separate wells at three to four different time points where one well is considered as one replicate. Two images were taken in each well and were used to calculate an average. CellProfiler (4.1.3) was used to quantify the number of PLA dots which were normalized to the nuclei count. Cytoplasm was determined by expanding the nuclei by 10 pixels in all directions. This area was used to remove PLA background in between cells. Antibody specificity: Antibodies were chosen based on knockout/knockdown validation in cell lines. This has been performed by the vendor, other scientific publications or by us.

### Actincytochemistry and image analysis

2 × 10^4^ WT/WT-derived MEFs and 1 × 10^4^ KO MEFs were seeded into 8-well chamber slides (Sigma Aldrich) and were rested o/n. Different numbers were seeded due to their difference in size and morphology. Cells were fixed in 4% paraformaldehyde (RT, 15 min) and permeabilized by 0.2% Triton-X100-PBS (RT, 15 min). Cells were blocked in 1% bovine serum albumin-PBS (RT, 60 min) and actin was stained using Alexa 488-conjugated Phalloidin (Thermofisher Scientific, RT, 45 min). DAPI was used to stain cell nuclei (RT, 5 min) before slides were mounted using ProLong gold antifade mountant (Thermofisher Scientific). Slides were imaged in an LSM 880 confocal microscope (Carl Zeiss, Jena, Germany) at 63× magnification. ZEN 2.3 SP1 FP3 (black) was used to obtain images. All conditions were run in 2 to 4 separate wells at three different time points where one well is considered as one replicate. Two images were taken in each well and were used to calculate an average. CellProfiler (4.1.3) was used to quantify the stained area, which was normalized to the nuclei count.

### Wound healing assay

MEFs were seeded into the inserts of a Culture-Insert 2 Well 24 plate (#80242, Ibidi). Due to the differences in cell size, 1.5 × 10^4^ *Hmgb1* knockout cells or 3 × 10^4^ *Hmgb1* wildtype cells (including transduced cells) were seeded into each well. Cells were rested o/n before removal of the inserts. Cells were imaged at 0 and 24 h in the Incucyte. Images were analyzed using the Wound healing size tool in ImageJ (36). Relative wound density was calculated using the width measurement. The experiment was performed in four wells, whereof an average was calculated and repeated at three different time points to obtain n = 3.

### Transwell assay

5 × 10^5^ THP-1 cells in RPMI supplemented with 1% fetal calf serum were seeded into the top chamber of a Corning HTS Transwell 96 well permeable supports. Cells migrated towards RPMI supplemented with 10% fetal calf serum. The cell number of the bottom chamber was determined after 5 h using Countess 3 (Invitrogen). In each experiment, technical triplicates were run and pooled before cell counting. The experiment was repeated at three separate time points to obtain n = 3.

### Statistical rationale

All data were normality tested using the D'Agostino & Pearson test or the Shapiro–Wilk test. Normally distributed data was analyzed by parametric tests. Other data were analyzed by nonparametric tests. *p*-values were corrected for multiple testing when comparing three groups or more. Details are found under the respective figure legend. Since data was obtained from cell lines, data points should be considered as technical replicates. Statistical analyses were performed in Graphpad Prism (9.5.1).

## Data availability

The mass spectrometry proteomics data have been deposited to the ProteomeXchange Consortium *via* the PRIDE (37) partner repository with the dataset identifier PXD060878. Processed and analyzed data can be found in data Table S1 and data Table S2.

## Supporting information

This article contains supporting information.

## Conflict of interest

The authors declare that they do not have any conflicts of interest with the content of this article.
