# Supplementary material for: Mapping the intracellular HMGB1 interactome and alterations induced by Toll-like receptor 4 activation
Source: J Biol Chem. 2025 Oct 27;301(12):110866. doi: 10.1016/j.jbc.2025.110866 (PMC12681554; doi:10.1016/j.jbc.2025.110866)
Supplement: Supporting Figures and Tables [file mmc1.pdf]

Mapping the intracellular HMGB1 interactome and alterations induced by Toll-like receptor 4 activation

Rebecka Heinbäck, Marit van der Zijde, Choi Har Tsang, Tilen Tršelič, Alexander Espinosa, Cecilia Aulin, Helena Erlandsson Harris

Supplementary information included:

- DNA sequence for MycBioID2-HMGB1
- Table S1
- Figure S1
- Table S2
- Table S3
- Table S4
- Figure S2
- Figure S3
- Figure S4

DNA sequence of MycBioID2-HMGB1

Clarification of highlights: Myc tag (blue) – BioID2 – linker (red) – HMGB1

ATGGAACAAAAACTCATCTCAGAAGAGGATCTCGACTTCAAGAACCTGATCTGGCTGAAGGAGGTG
GACAGCACCCAGGAGAGACTGAAGGAGTGGAACGTGAGCTACGGCACCGCCCTGGTGGCCGACAGAC
AGACCAAGGGCAGAGGCGGCCTGGGCAGAAAGTGGCTGAGCCAGGAGGGCGGCCTGTACTTCAGCTT
CCTGCTGAACCCCAAGGAGTTCGAGAACCTGCTGCAGCTGCCCCTGGTGCTGGGCCTGAGCGTGAGCG
AGGCCCTGGAGGAGATCACCGAGATCCCCTTCAGCCTGAAGTGGCCCAACGACGTGTACTTCCAGGAG
AAGAAGGTGAGCGGCGTGCTGTGCGAGCTGAGCAAGGACAAGCTGATCGTGGGCATCGGCATCAACG
TGAACCAGAGAGAGATCCCCGAGGAGATCAAGGACAGAGCCACCACCCTGTACGAGATCACCGGCAA
GGACTGGGACAGAAAGGAGGTGCTGCTGAAGGTGCTGAAGAGAATCAGCGAGAACCTGAAGAAGTTC
AAGGAGAAGAGCTTCAAGGAGTTCAAGGGCAAGATCGAGAGCAAGATGCTGTACCTGGGCGAGGAG
GTGAAGCTGCTGGGCGAGGGCAAGATCACCGGCAAGCTGGTGGGCCTGAGCGAGAAGGGCGGCGCCC
TGATCCTGACCGAGGAGGGCATCAAGGAGATCCTGAGCGGCGAGTTCAGCCTGAGAAGAAGC**CTCGA**
GGGTGGAGGCGGGTCTATGGGCAAAGGAGATCCTAAGAAGCCGAGAGGCAAAATGTCATCATATGC
ATTTTTTGTGCAAACTTGTCGGGAGGAGCATAAGAAGAAGCACCCAGATGCTTCAGTCAACTTCTCAG
AGTTTTCTAAGAAGTGCTCAGAGAGGTGGAAGACCATGTCTGCTAAAGAGAAAGGAAAATTTGAAGA
TATGGCAAAAGCGGACAAGGCCCGTTATGAAAGAGAAATGAAAACCTATATCCCTCCCAAAGGGGAG
ACAAAAAAGAAGTTCAAGGATCCCAATGCACCCAAGAGGCCTCCTTCGGCCTTCTTCCTCTTCTGCTCT
GAGTATCGCCCAAAAATCAAAGGAGAACATCCTGGCCTGTCCATTGGTGATGTTGCGAAGAAACTGGG
AGAGATGTGGAATAACACTGCTGCAGATGACAAGCAGCCTTATGAAAAGAAGGCTGCGAAGCTGAAG
GAAAAATACGAAAAGGATATTGCTGCATATCGAGCTAAAGGAAAGCCTGATGCAGCAAAAAAGGGAG
TTGTCAAGGCTGAAAAAAGCAAGAAAAAGAAGGAAGAGGAGGAAGATGAGGAAGATGAAGAGGATG
AGGAGGAGGAGGAAGATGAAGAAGATGAAGATGAAGAAGAAGATGATGATGATGAATAA

Table S1. Primer pairs used in qPCR.

Gene symbol	Forward primer	Reverse primer
Human *HMGB1*	5’-TACGAAAAGGATATTGCTGC	5’-CTCCTCTTCCTTCTTTTTCTTG
Human *GAPDH*	5’-TCGGAGTCAACGGATTTG	5’-CAACAATATCCACTTTACCAGAG
Mouse *Hmgb1*	5’-CGCGGAGGAAAATCAACTAA	5’-TCATACGAGCCTTGTCAGC
Mouse *Hprt*	5’-AGGGATTTGAATCACGTTTG	5’-TTTACTGGCAACATCAACAG

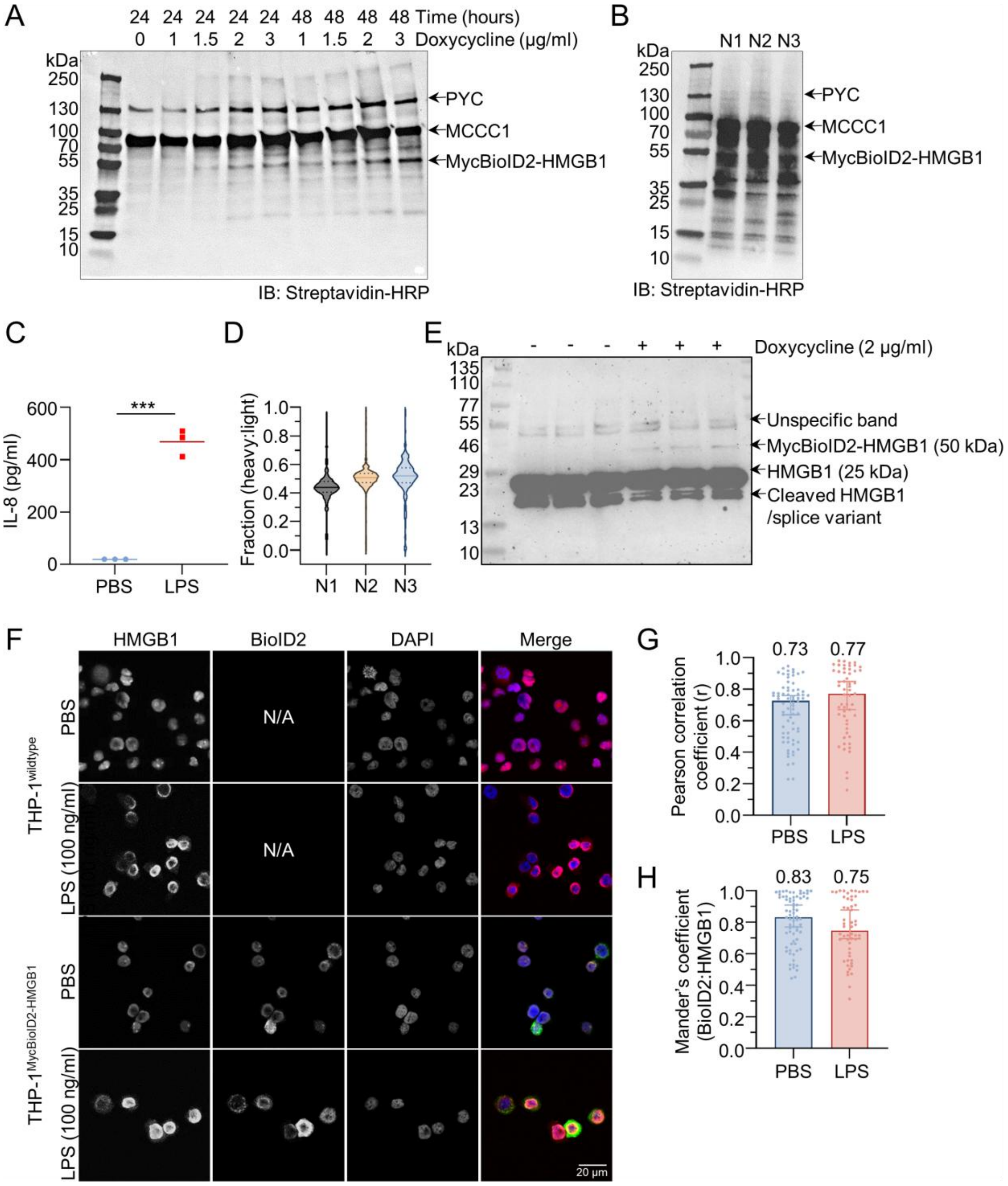

A
Time (hours) 24 24 24 24 24 48 48 48 48
Doxycycline (µg/ml) 0 1 1.5 2 3 1 1.5 2 3
kDa 250 130 100 70 55 35 25 15 10
PYC
MCCC1
MycBioID2-HMGB1
IB: Streptavidin-HRP
B
N1 N2 N3
kDa 250 130 100 70 55 35 25 15 10
PYC
MCCC1
MycBioID2-HMGB1
IB: Streptavidin-HRP
C
IL-8 (pg/ml)
***
PBS LPS
D
Fraction (heavy:light)
N1 N2 N3
E
Doxycycline (2 µg/ml) - - - + + +
kDa 135 110 77 55 46 29 23 13 10
Unspecific band
MycBioID2-HMGB1 (50 kDa)
HMGB1 (25 kDa)
Cleaved HMGB1 /splice variant
F
HMGB1 BioID2 DAPI Merge
THP-1wildtype
THP-1MycBioID2-HMGB1
PBS
LPS (100 ng/ml)
N/A
20 µm
G
Pearson correlation coefficient (r)
0.73 0.77
PBS LPS
H
Mander's coefficient (BioID2:HMGB1)
0.83 0.75
PBS LPS

Figure S1. A. THP-1$^{MycBioID2\text{-}HMGB1}$ cells were treated with different doxycycline for 24 or 48 hours followed by treatment with biotin for 24 hours. Lysates were analyzed by Western blot against biotinylation using streptavidin-HRP. **B.** After streptavidin purification of the lysates used for BioID, samples were analyzed by Western blotting using streptavidin-HRP. All samples showed enrichment of biotinylated proteins. Expected sizes of MycBioID2-HMGB1 and the endogenously biotinylated proteins PYC and MCCC1 are marked with arrows. **C.** IL-8 levels in the THP-1 supernatants (n=3) from the cells used for BioID. Data is shown individual data points and was analyzed by Student's t-test. *** is equivalent to a p-value < 0.001. **D.** The heavy:light ratios of all proteins in each sample analyzed by LC-MS/MS. The theoretical value should be 0.5 since the light and heavy samples were pooled 1:1 before LC-MS/MS. **E.** THP-1$^{MycBioID2\text{-}HMGB1}$ cells were treated with different 2 µg/ml doxycycline 48 hours followed and lysates were analyzed by Western blot against HMGB1 to detect both endogenous and MycBioID2-HMGB1. **F**. Immunocytochemistry of HMGB1 (red) and BioID2 (green) in resting (n=3) and LPS stressed (n=3) wildtype and MycBioID2-HMGB1-expressing THP-1 cells. Scale bar represents a distance of 20 µm. **G.** The stained cells were analyzed by pixel-by-pixel correlation using Pearson correlation tests on resting (n=71) and LPS stressed (n=55) cells. Each data point represent one cell and data is shown as individual data points and the median ± 95% confidence interval. **H.** The fraction of MycBioID2-HMGB1 that overlaps with endogenous HMGB1 was quantified using Mander's overlap coefficient on resting (n=71) and LPS stressed (n=55) cells. Each data point represent one cell and data is shown as individual data points and the median ± 95% confidence interval. .

Table S2. Summary of proteins analyzed by *in silico* HMGB1-protein docking. *Proteins with acceptable docking models and confirmed by proximity ligation assays.

Protein	Full name	Described functions
ACIN1	Apoptotic chromatin condensation inducer in the nucleus	Induces apoptotic chromatin condensation. Part of the splicing-dependent multiprotein exon junction complex.
CACTIN	Cactin	Involved in pre-mRNA splicing. Negative regulator of TLR signaling.
*CAT	Catalase	Enzyme that catalyzes the degradation of H_2O_2.

DVL1	Dishevelled-1	Regulates Wnt signaling and is directly downstream of Frizzled receptors.
*DVL2	Dishevelled-2	Regulates Wnt signaling and is directly downstream of Frizzled receptors.
*GNA12	Guanine nucleotide-binding protein subunit alpha-12	G-protein coupled receptor signaling. Involved in Rho signaling
*HCLS1	Hematopoietic lineage cell-specific protein	Regulate ARP2/3 polymerization as an adaptor protein. Only expressed in hematopoietic cells.
*HSPB1	Heat shock protein B1	Belongs to the family of small heat shock proteins. Acts as a chaperone.
KOW domain-containing protein	KOW domain-containing protein	Unknown
MTHFD1L	Monofunctional C1-tetrahydrofolate synthase, mitochondrial	Unknown but predicted to be involved in one-carbon folate metabolism
*NIFK	MKI67 FHA domain-interacting nucleolar phosphoprotein	Regulates proliferation
RAB1A	Ras-related protein Rab-1A	Small GTPase regulating vesicular transport from the endoplasmic reticulum to Golgi. Have a role in autophagosome formation.
*S100A6	Protein S100-A6	Reported to have various roles including cytoskeletal function, stress response, cell proliferation, cell differentiation, and signal transduction
TADA2B	Transcriptional adapter 2-beta	Transcriptional adaptor protein by coordinating histone acetyltransferase
TPD52L2	Tumor protein D54	Reported to associate to cell membrane and vesicles
TTC1	Tetratricopeptide repeat protein 1	Reported to bind Gα proteins and Ras
*NR3C1	Glucocorticoid receptor	Known HMGB1 interactor used as positive in our experiments. Nuclear receptor activated by glucocorticoids.

MD2	Lymphocyte antigen 96	Known HMGB1 interactor used as comparator in docking models. Facilitates LPS binding to TLR4.

Table S3. RMSD scores of all docked HMGB1-protein interactions.

	PROTEINS	I_{RMS} (Å)	L_{RMS} (Å)	*f*(nat)	CAPRI RANKING
Increased by LPS	KOW domain-containing protein	0.09	1.25	0.58	High
	GNA12	2.43	8.30	0.35	Acceptable-Medium
	TTC1	3.61	15.10	0.19	Unacceptable
	TADA2B	2.27	11.19	0.6	Unacceptable
	TPD52L2	13.54	15.69	0.27	Unacceptable
	ACIN1	2.13	10.21	0.20	Unacceptable
	RAB1A	2.97	6.48	0.09	Unacceptable
	MTHFD1L	1.54	11.93	0.37	Unacceptable
No abundance change	HCLS1	0.25	1.81	0.39	Medium-High
	S100A6	0.10	5.29	0.72	Medium
	DVL2	9.59	2.90	0.57	Acceptable
	DVL1	10.88	2.07	0.29	Unacceptable
Decreased by LPS	HSPB1	2.01	2.02	0.9	Medium
	NIFK	0.56	3.40	0.76	Medium-High
	CAT	2.57	7.67	0.29	Acceptable
	CACTIN	3.59	14.54	0.4	Unacceptable
Comparison to known HMGB1 protein interactions	NR3C1	1.98	5.32	0.79	Medium
	MD2	1.24	3.20	0.81	Medium-High

I_{RMS} - The root mean square deviation (RMSD) of amino acids at the interface. L_{RMS} – The overall RMSD of entire complex. F(nat) – the fraction of correct contacts at the interface (within 5 Å). CAPRI ranking is the official ranking system summarizing all three values ranging from unacceptable – acceptable – medium – high.

Table S4. Table summarizing the top five interacting amino acids in HMGB1 and its interactor of all acceptable docking models.

	PROTEIN	TOP 5 INTERACTING AMINO ACIDS -HMGB1	TOP 5 INTERACTING AMINO ACIDS -INTERACTOR	CAPRI RANKING
**INCREASED BY LPS**	KOW domain-containing protein	Asp207<br>Glu199<br>Phe38<br>Glu206<br>Glu198	Arg45<br>Arg59<br>Lys2<br>Phe3<br>Lys103	High
	GNA12	Phe38<br>Glu199<br>Glu47<br>Ser42<br>Glu56	Arg6<br>Ser9<br>Leu47<br>Arg51<br>Asp243	Acceptable-Medium
**NO ABUNDANCE CHANGE**	HCLS1	Phe103<br>Arg110<br>Arg97<br>Pro99<br>Lys96	Tyr481<br>Tyr437<br>Glu442<br>Asp438<br>Glu446	Medium-High
	S100A6	Phe103<br>Arg97<br>Phe102<br>Lys88<br>Lys82	Phe70<br>His27<br>Val13<br>His17<br>Gln71	Medium
	DVL2	Tyr16<br>Arg10<br>Met1<br>Lys76<br>Arg73	Arg442<br>Arg440<br>Tyr393<br>Phe390<br>Gly388	Acceptable
**DECREASED BY LPS**	HSPB1	Phe103<br>Pro118<br>Arg110<br>Lys87<br>Lys114	Tyr54<br>Phe29<br>Trp51<br>Leu35<br>Gln31	Medium
	NIFK	Glu194<br>Asp211<br>Glu195<br>Ser107<br>Glu191	Lys213<br>Lys214<br>Lys212<br>Lys215<br>Arg211	Medium-High

	CAT	Ile122<br>Phe103<br>Arg110<br>Ala126<br>Ser107	Met61<br>Asp65<br>Phe57<br>Leu50<br>Glu60	Acceptable
**COMPARISON TO KNOWN HMGB1 PROTEIN INTERACTIONS**	NR3C1	Tyr16<br>Phe38<br>Met1<br>Arg24<br>Val20	Lys495<br>Lys442<br>Glu221<br>Glu231<br>Asp220	Medium
	MD2	Phe38<br>Tyr16<br>Val20<br>Ala17<br>Ser14	Phe7<br>Phe11<br>Phe4<br>Ser8<br>Phe15	Medium-High

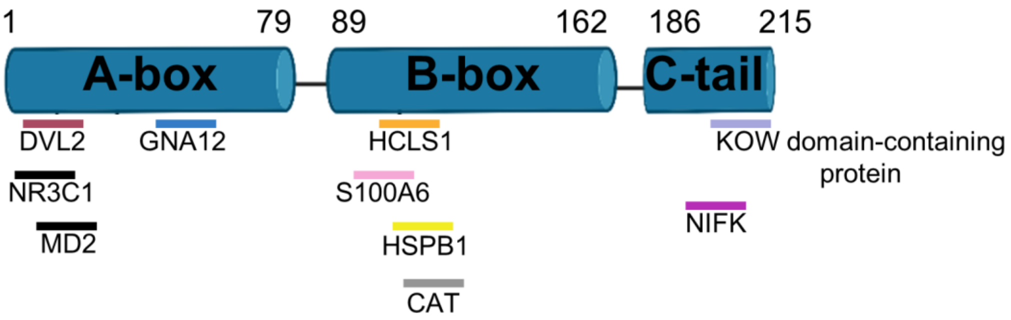

Figure S2. Illustrates what domain of HMGB1 the different interactors are predicted to bind.

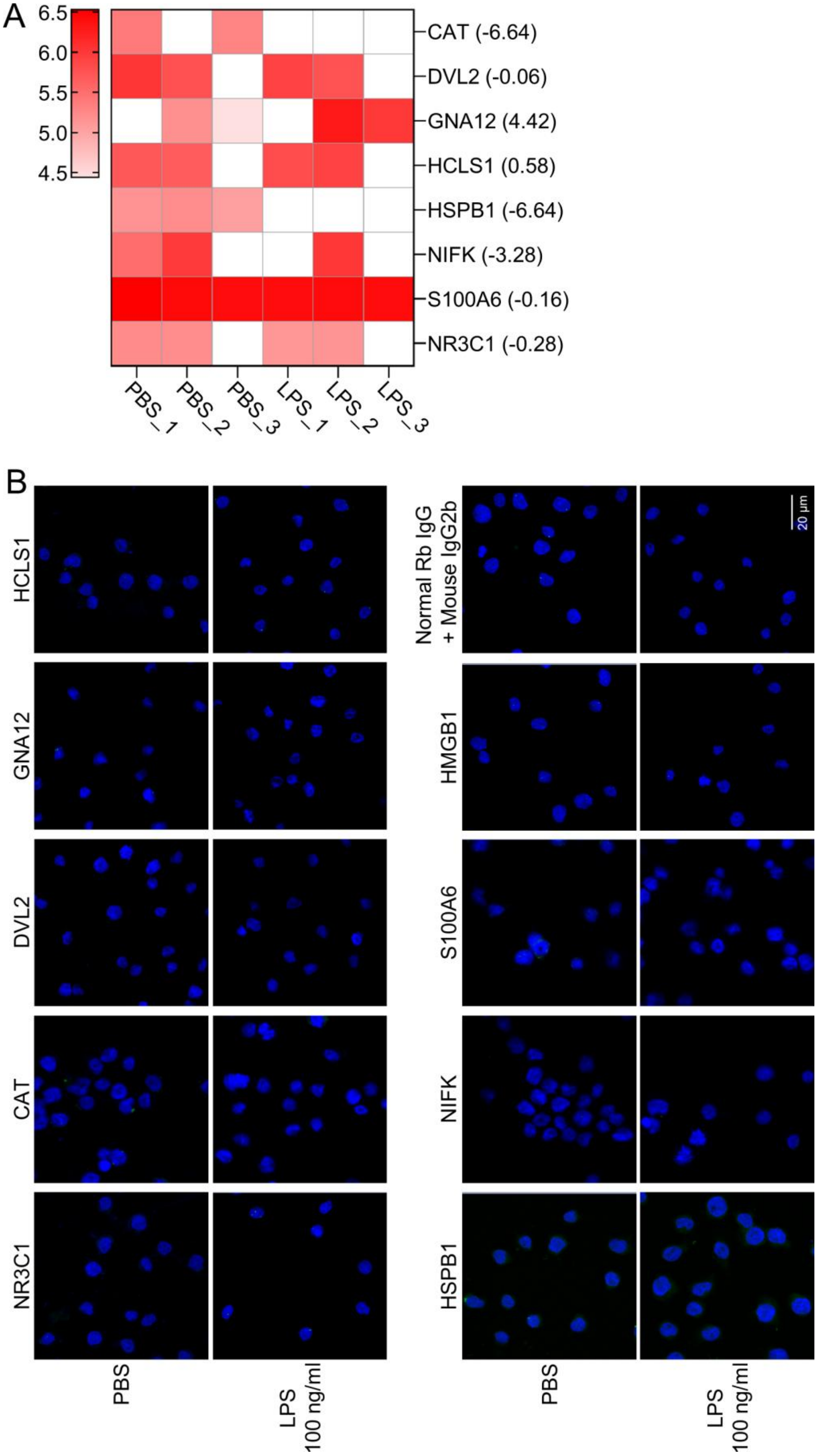
A
6.5
6.0
5.5
5.0
4.5
CAT (-6.64)
DVL2 (-0.06)
GNA12 (4.42)
HCLS1 (0.58)
HSPB1 (-6.64)
NIFK (-3.28)
S100A6 (-0.16)
NR3C1 (-0.28)
PBS_1
PBS_2
PBS_3
LPS_1
LPS_2
LPS_3
B
HCLS1
GNA12
DVL2
CAT
NR3C1
Normal Rb IgG + Mouse IgG2b
HMGB1
S100A6
NIFK
HSPB1
20 μm
PBS
LPS 100 ng/ml
PBS
LPS 100 ng/ml

Figure S3. A. The LC-MS/MS protein abundances (log_{10}), obtained by BioID, of the targets later analyzed by PLA were put into a heatmap. The quantified log_2fold changes are showed in brackets. **B.** Representative images of all negative controls performed in the proximity ligation assays including stainings using single antibodies or normal rabbit IgG and mouse IgG2b. Scale bar represents a distance of 20 µm.

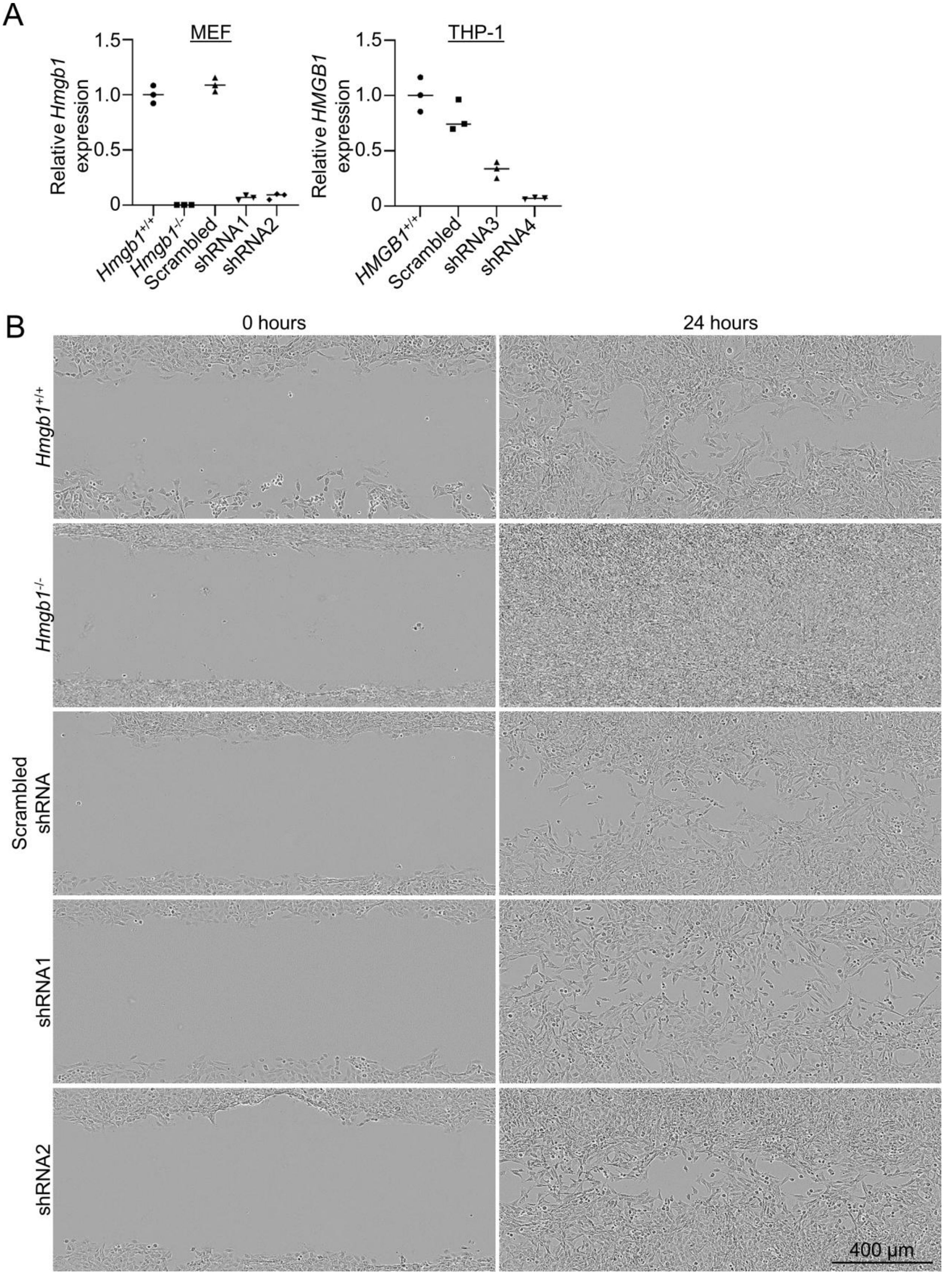
A
MEF
THP-1
Relative *Hmgb1* expression
Relative *HMGB1* expression
1.5
1.0
0.5
0
Hmgb1+/+
Hmgb1-/-
Scrambled
shRNA1
shRNA2
HMGB1+/+
shRNA3
shRNA4
B
0 hours
24 hours
Hmgb1+/+
Hmgb1-/-
Scrambled shRNA
shRNA1
shRNA2
400 μm

Fig S4. A. The degree of *Hmgb1* knockout and shRNA-induced knockdown in MEFs and THP-1 cells was quantified by qPCR. Mouse data was normalized against *Hprt* and human data was normalized against *GAPDH.* Data is shown as fold change against wildtype cells. **B.** Uncropped source images for the data presented in Figure 5A. Shows wounds at 0 and 24 hours after insert removal. *Hmgb1* wildtype and knockout MEFs were used. Also, inducible knockdown in wildtype MEFs was done using different *Hmgb1*-targeting shRNA construct and scrambled shRNA control. Scale bar represents a distance of 400 µm.
